# Genevan encounters with Newton. Gabriel Cramer, Jean-Louis Calandrini and the annotated edition of the *Principia*

**DOI:** 10.1098/rsta.2023.0277

**Published:** 2025-07-17

**Authors:** Philip Beeley

**Affiliations:** ^1^ History Faculty, University of Oxford, Oxford, England, UK

**Keywords:** Gabriel Cramer, Alexis Clairaut, Isaac Newton, theory of the Moon, apsides, Geneva edition

## Abstract

The named editors of the annotated edition of Newton’s *Principia*, François Jacquier and Thomas Le Seur, saw their painstaking enterprise as a decisive means to making the great polymath’s work more accessible. As this article makes clear, the Genevan edition was above all a collaborative enterprise, resting crucially on the contributions of the two Genevan mathematicians Jean-Louis Calandrini and Gabriel Cramer, both of whose expertise complemented and, in some respects, exceeded that of the two Minim priests based in Rome, while also being attuned to contemporary scientific discussion involving the foremost scientific figures of the time, such as Euler, Clairaut, d’Alembert and the Bernoulli. Focusing in particular on the debate over Newton’s theory of the Moon, the article reveals the importance of Calandrini’s and Cramer’s scientific networks for understanding the production and reception of the Geneva edition.

This article is part of the theme issue ‘Newton, *Principia*, Newton Geneva Edition (17th–19th) and modern Newtonian mechanics: heritage, past & present’.

## 1. Introduction

For the Swiss mathematician Leonhard Euler (1707−1783), it was fortunate that escalating violence and political turmoil in Russia in 1741, following the death of Empress Anna the previous year, coincided with an invitation from Frederick II of Prussia to assist in reconstructing the Academy of Sciences in Berlin.^1^ Such was Euler’s relief after his safe arrival on the Spree that he immediately wrote to his former colleague in St Petersburg, the mathematician and cryptographer Christian Goldbach (1690−1764), describing at length his arduous journey but also expressing his understandable concern for the transportation to Germany of his personal belongings and the payment of his outstanding professorial salary.^2^

In his short reply, Goldbach addressed Euler’s concerns, asked his opinion on a theorem in algebra, and cited an interesting piece of news he had recently come across in a scholarly journal:

In the *Zeitungen von gelehrten Sachen* I read a short time ago that the two monks who are editing Newton’s *Principia mathematica* have made extensive use of your *Mechanica*.^3^

The journal to which Goldbach refers was a sister publication of the more famous *Acta eruditorum* and could claim to have a common publisher and editor, Friedrich Otto Mencke (1708−1754), son of the *Acta*’s founder, Otto Mencke (1644−1707). Having a chief focus on scholarly news and reviews of recent publications, the *Neue Zeitungen von Gelehrten Sachen* for the year 1741 had devoted a considerable part of its 17 July issue to a detailed and suitably informed description of the second volume of the Geneva edition of Isaac Newton’s (1643−1727) *Principia*, published the previous year by the printer and bookseller Barrillot and son.^4^ The length of the review was probably intended to compensate for the fact that the journal had failed to review the first volume of the Genevan enterprise. Having pointed out the endeavour of Thomas Le Seur (1703−1770) and François Jacquier (1711−1788) to render through their extensive annotations Newton’s work ‘so clear that it can be understood by a reader who has only moderate knowledge of geometry’,^5^ the unnamed reviewer addresses the question of the sources employed by the two Minim priests in a passage that evidently caught Goldbach’s eye:

[Le Seur and Jacquier] in their annotations not only explain Newton’s propositions, but also set out other discoveries. They have indicated what is of relevance hereunto contained in the Acta eruditorum, the proceedings of the Paris and Petersburg academies, and in Hermann’s *Phoronomia*. Euler’s *Mechanica* has been particularly strongly cited by them. They have also benefitted considerably from certain works of Poleni. In some cases the authors include the discoveries of other mathematicians […], while in others they simply refer the reader to the writings concerned where something is explained.^6^

Indeed, the two ostensible editors and the Genevan mathematician Jean-Louis Calandrini (1703−1758), who was largely responsible for giving the three-volume edition its final form and seeing it through the press,^7^ drew extensively on contemporary publications in their notes and commentaries, including besides the aforementioned Jacob Hermann (1678−1733), Euler and Giovanni Marchese Poleni (1683−1761), along with Newton’s British commentators, such as James Stirling (1692−1770), the Dutch mathematician Willem ’s Gravesande (1688−1742) and diverse members of the Bernoulli family. But how was the Genevan Newton edition for its part viewed by contemporary scholars such as these, and what can we learn from members of scientific circles in the Genevan Republic about how the work itself came about? A key figure whose letters and writings throw important light on these and other questions is another Genevan mathematician and a close friend of Calandrini’s, namely Gabriel Cramer (1704−1752).^8^

## 2. Cramer and the Bernoulli

A member of a well-established Genevan family with strong ties to the world of publishing, Gabriel Cramer is known for having been one of the candidates alongside Amédée de la Rive (1692−1763) and Calandrini for the chair in philosophy at the Académie de Genève following the death of the previous incumbent Étienne Jallabert (1658−1723). In the end, De la Rive was successful, but Cramer and Calandrini so impressed the city’s governing council that it was decided to create a new chair in mathematics and award it jointly to these two young friends.^9^ The rationale of this decision was quite simply that in addition to his other duties Jallabert had previously given instruction in mathematics as well.^10^ Nicknamed Castor and Pollux on account of their perceived inseparability, Cramer and Calandrini soon formed an arrangement whereby in rotation one would carry out teaching duties in Geneva while the other furthered his knowledge by travelling. They also divided up teaching responsibilities amicably, with Cramer focusing on geometry and mechanics, while Calandrini taught algebra and astronomy.^11^ Thanks to their arrangement, Cramer was able to follow up a 2-year stint of teaching by travelling, in early 1727, to Basel, where he became a pupil of Johann I Bernoulli (1667−1748). In a long letter to Calandrini, sent in May that year, Cramer describes the programme of study he was following:

I go regularly every day to M. Johann Bernoulli and almost as often to his nephew Nicolas Bernoulli. […] I converse principally with the former on differential and integral calculus; he has conveyed to me his manuscript lessons which he gave to the Marquis de l’Hospital.^12^

From this letter, we learn that Cramer also met regularly with Nicolaus I Bernoulli (1687−1759), who likewise worked on differential equations, but above all was occupied with questions of probability theory and games of chance. In his youth, Nicolaus had studied mathematics under Jacob Bernoulli (1655−1705) and during that time had been able to see drafts of his uncle’s *Ars conjectandi*, in the publication of which he would later be crucially involved. Animated by what he found in those papers, Nicolaus had embarked upon an academic peregrination of Europe in 1712, with the aim of extending his knowledge in fields that interested him. In the course of his tour, he had met and discussed probability problems with Pierre Rémond de Montmort (1678−1719) in Paris and Abraham de Moivre (1667−1754) in London, both men ultimately having a profound influence on his intellectual development.^13^ It is highly likely that Cramer’s itinerary on his extended tour through Europe was at least in part influenced by Nicolaus Bernoulli’s accounts of his own experience. Like Nicolaus, Cramer, too, would undertake visits to London and Paris to immerse himself in the scientific circles that flourished in those cities.

Although Cramer stayed in Basel just a few months, from May until October 1727, his time there effectively launched his scientific career. Johann I Bernoulli’s contribution was decisive, because he enabled Cramer to start building up his own network of scientific contacts and correspondents. Gaining a good reputation was important in this respect. With Bernoulli’s help, Cramer slowly but surely succeeded in establishing himself as a promising and, despite his youth, respected member of the Republic of Letters. After Cramer had departed from Basel, Bernoulli wrote to his friend, the Genevan medical practitioner Daniel Leclerc (1652−1728), providing an account of the positive impression the visitor had made on him:

I am very happy to have made the personal acquaintance of M. Cramer; he did me the honour of attending my lessons on the geometry of infinites. I have already been able to glimpse something of the knowledge and ability he possesses, as young as he is. I foresee for him that he will become a great and excellent mathematician.^14^

## 3. Cramer’s *Peregrinatio Academica*

Cramer’s European tour, which would last from 1727 to 1729, took him initially to Paris with a suitably upbeat letter of introduction from Johann I Bernoulli to the eminent mathematician and natural philosopher Jean Jacques d’Ortous de Mairan (1678−1771) praising the young scholar’s ability. A suitable reception at the Académie des Sciences in the immediate future was thus ensured with the promise, sketched out in the planned itinerary, that Cramer would return later for a prolonged stay, during which he would pursue further his scientific interests:

The carrier of this letter by the name of M. Cramer is professor of mathematics in Geneva. He is a man of great merit and a very skilled mathematician, although he is still very young. Having received permission from his governors to travel, he has made his first stay here for about five months with the aim of benefitting somewhat from my scant knowledge […] After leaving England, he will return to Paris so as to stay there some time, his plan being without doubt to become acquainted with distinguished scholars and to carry out astronomical observations. ^15^

From the French capital, Cramer proceeded to London, where with the support of the Royal Society’s secretary James Jurin (1684−1750), he not only attended meetings of that institution, but also became acquainted with some of the most illustrious scientific figures members in the metropolis as well as at the universities of Oxford and Cambridge, including Edmond Halley (1656−1742), John Machin (c. 1686−1751), de Moivre, Nicholas Saunderson (1682−1749) and James Stirling.^16^ Meeting with learned men and women and broadening one’s scientific interests was after all the main reason for undertaking such journeys in the first place. There was one concrete result in particular: if up to this time he had occupied himself principally with the mathematics of Gottfried Wilhelm Leibniz (1646−1716), his sojourn in England signified for Cramer the beginning of his lifetime engagement alongside the German mathematician, also with the work of Newton.

By July 1728, Cramer was in Leiden, where he became acquainted with Willem Jacob ’s Gravesande , one of the first scholars to introduce Newtonian ideas on the Continent.^17^ He later reports having had numerous discussions with ’s Gravesande on questions relating to the theory of motion and specifically on potential implications of Leibniz’s concept of the conservation of *vis viva*, a hotly debated topic at that time.^18^

Unfortunately, however, Cramer’s sojourn in the Low Countries was marred by a long period of illness, and it was not until the beginning of the following year, by which time he had arrived in Paris, that he was able to write to Jurin, thanking him for his kindness during his stay in England. Much of his letter is taken up with deliberations on *vis viva* and the theory of motion, but towards the end, when turning to items of mathematical news, Cramer remarks

Some body told me in England that Mr. Machin shou’d publish very soon a Treatise about the Theory of the Moon. Is there nothing of it publish, or under the press? Is nothing printed of new, of Sir Isaac’s precious remains?^19^

Although it would be some time before new works of Newton would be published posthumously, in that year, 1729, Andrew Motte’s (1696−1734) English translation of the *Principia* came out in an edition that also included Machin’s attempt at rectifying imperfections in Newton’s lunar theory as an appendix.^20^ While John Machin is justly honoured for the series expansion of π he produced, his *Laws of the Moon’s Motion* is unfortunately noted for its failures.

## 4. Paris and thereafter

Nicolaus Bernoulli was clearly informed of Cramer’s travel plans and knew that he would be spending a considerable time in the French metropolis. Following a common practice in the Republic of Letters, Nicolaus therefore took the opportunity of the departure of a fellow mathematician from Basel en route for Paris to catch up on his correspondence with his Genevan friend. His letter is remarkable because he uses it to introduce Cramer to the bearer, the learned Swedish mathematician Samuel Klingenstierna (1698−1765), who for his part was keen to make the acquaintance of the young Genevan about whom he already had a high estimation based on the favourable portraits provided both by Nicolaus and his uncle Johann I Bernoulli.^21^ Klingenstierna’s keen interest in the scientific work of Newton—a topic on which he lectured—led to his noted discovery of errors in Newton’s theory of refraction.

Evidently keen to promote Cramer’s mathematical studies, Nicolaus Bernoulli suggested that he take a look at Klingenstierna’s ‘ingenious’ although rather long method for solving the problem of linear recurrent sequences, noting that this problem had also been solved by his cousin, Daniel Bernoulli (1700−1782) in a paper presented to a meeting of the St Petersburg Academy of Sciences, a copy of which Daniel had sent to him from Russia.^22^

Following his short stay in Holland, Cramer arrived back in Paris in December 1728 and remained there until May the following year. He was already well acquainted with Mairan, but now through attending various salons and public meetings of the Paris Académie, he was able to extend his circle of acquaintances further to include among others Bernard le Bovier de Fontenelle (1657−1757), Pierre Louis Moreau de Maupertuis (1698−1759), René-Antoine Ferchault de Réaumur (1683−1757) and the mathematical prodigy Alexis Claude Clairaut (1713−1765).^23^ Numerous letters he subsequently exchanged with Mairan and Clairaut reveal that already at this time Cramer shared their interest in the properties of algebraic curves and particularly the problem of maxima and minima.^24^ It would however be another two decades before Cramer published a major work on this topic. In the meantime, as we shall see, a rather different issue became a focus of their attention: the problem of deriving an accurate account of lunar motion.

Following his return to Geneva in May, Cramer occupied himself primarily with questions on mechanics and astronomy. On the back of these investigations, he submitted in the following year a response to the prize question of the Paris Académie seeking the best explanation for why the planets move in elliptical orbits. The precise terms in which the academicians raised the question was ‘What is the cause of the elliptical figure of planetary orbits, and why does the major axis of these ellipses change its position, or what amounts to the same, why does their aphelion or their apogee correspond successively to different points in the sky?’^25^ The prize was eventually awarded to Johann I Bernoulli, a result which no doubt can be put down to the fact that Bernoulli in his submission remained faithful to the vortex theory of René Descartes (1596-1650). The *Mémoire* submitted by Cramer, under a motto drawn from Psalm 138—‘The darkness is to him just as the lightness’—was awarded *proxime accessit*.^26^ It is a measure of Cramer’s loyalty to his former teacher that he would subsequently describe it as an honour to have been defeated by Johann I Bernoulli.^27^

Most of Cramer’s days in Geneva were now dedicated to teaching. After Calandrini had taken up the better remunerated and less demanding chair in philosophy at the Académie de Genève, he was alone responsible for providing instruction in mathematics.^28^ Alongside teaching, he devoted himself to a truly prodigious amount of scientific editing.^29^ Between 1732 and 1741, he brought out with the Lausanne and Genevan publisher Marc-Michel Bousquet (1696−1762) the five volumes of Christian Wolff’s (1679−1754) *Elementa matheseos universae* enriched with his own commentaries and annotations.^30^ In 1742 Bousquet published the four-volume *Opera omnia* of Johann I Bernoulli, likewise with his commentaries and annotations, Cramer having been solicited to produce the edition both by the publisher and Johann II Bernoulli (1710−1790).^31^ This was followed 2 years later by the two-volume edition of the works of Jacob Bernoulli, this time with the Genevan publishing house owned by his cousins Gabriel (1723−1793) and Philibert Cramer (1727−1779) together with Claude Philibert (1736−1784).^32^ In 1745, Cramer together with Jean Castillon (born: Giovanni Salvemini di Castiglione, 1708−1791) edited the two-volume *Commercium philosophicum et mathematicum* of Leibniz and Johann I Bernoulli, this work being published by Bousquet and serving as an important source of Leibniz’s mathematics for over a century.^33^

## 5. Clairaut and the theory of the Moon

During this intellectually productive period, Cramer conducted an intensive correspondence with Clairaut in Paris. Not only did their scientific interests often coincide, especially on questions of algebraic curves, but they also regularly exchanged publications, including in Cramer’s case some of the scientific works he had edited. Although their letters touched on a variety of topics, their correspondence became focused, from the spring of 1644 onwards, on contemporary efforts in Paris, including those of Clairaut himself, to establish precisely the mathematical physics of the Moon’s orbit. In the course of his investigations, Clairaut like Maupertuis, Euler and Jean-Baptiste le Rond d’Alembert (1717−1783), had reached the same—as it happens erroneous—conclusion that Isaac Newton’s law on universal gravitation was inadequate and needed to be revised.^34^ Put differently, all four men were convinced that Newton’s inverse square law could quite simply not be brought into agreement with empirically observable phenomena of the lunar orbit. Nor was this a matter of closed intellectual discourse conducted within an elite circle of scholars. Clairaut set out his conclusion that Newton’s force law was inadequate and needed to be corrected at the meeting of the Académie des Sciences in Paris on 15 November 1747. This was the first meeting of the new academic year that was traditionally open to the public. The occasion could not have been more opportune—so it must have seemed to Clairaut—for setting out his momentous claim on the veracity of the *Principia*.

Seated among the audience on that occasion was Gabriel Cramer. Fortune would have it that the Genevan mathematician had taken on an engagement to accompany Frederick-Louis, hereditary prince of Saxe-Gotha-Altenburg (1735−1756) to Paris as part of the young nobleman’s Grand Tour.^35^ Cramer stayed there with his charge from April to November, while Frederick-Louis himself remained for some 3 years under the supervision of Baron Ulrich von Thun (1707−1788), master of the royal household. During that time, Frederick-Louis accompanied von Thun to various Parisian salons and by this means was introduced to such leading Enlightenment figures as Voltaire (1694−1778), Jean-Jacques Rousseau (1712−1778), Baron d’Holbach (1723−1789) and Denis Diderot (1713−1784).

In the course of the *Mémoire* he presented at the Académie, Clairaut explained that reconciling observations of the eccentricity of the lunar orbit, specifically the precession of the apsides, with the system delineated in the *Principia* required treating the Earth, the Sun and the Moon as an instance of the three-body problem, which had gained traction in recent years through developments in infinitesimal calculus brought about by Gottfried Wilhelm Leibniz and the brothers Johann and Jacob Bernoulli.^36^ Part of the problem in Newton’s theory was his treatment of the ‘tidal’ action of the Sun in perturbing the motion of the Moon in relation to the Earth, particularly as set out in corollary 2 of proposition 45, Book I, and propositions 26−29, Book III. When Newton substituted his average ratio of what he called the ‘foreign force’, i.e. the radial component of the Sun’s perturbing force to the centripetal force in his theoretically developed formular, it yielded only about half of the average observed motion of the Moon’s apogee per revolution. Famously, Newton himself inserted the line after his calculation in Book I, ‘The apse of the motion is about twice as swift’.^37^

As Clairaut recognized, Newton had only been able to deliver an incomplete account of the motion of the apsides, i.e. the line connecting the apogee or point at which the Moon is furthest from the Earth, and the perigee, the point at which it is nearest. While he assumed the empirical value of the lunar apse of 3°4′11″ to be correct, he found that only about half this value could be derived on the basis of Newton’s inverse-square law. In consequence, Clairaut proposed modifying the inverse-square law by the addition of an inverse fourth power term in order to make up the difference.^38^

By all accounts, during the lively discussion that ensued following Clairaut’s presentation, Cramer pointed out that his Genevan friend Calandrini had discovered the weakness in Newton’s theory earlier, but through his inherent modesty had chosen not to publish his result.^39^ The public exchanges at the Académie were based, therefore, on scientific facts that both Clairaut and Cramer were privately well aware of already. Moreover, Clairaut had known for some time of Calandrini’s work on the topic, although he had been unable to elicit anything substantial in writing from him. In other words, Clairaut’s 1747 *Mémoire* represents the initial public culmination of an essentially private debate that had already been underway for a considerable time.

Thus, in a letter to the French mathematician written a good three and a half years earlier, in March 1744, Cramer recalled that in reading the *Principia* what had appeared to him most difficult was the calculation of the movement of the Moon at its apogee that seemed to him not to agree well with published tables calculated on the basis of John Flamsteed’s (1646−1719) observational data.^40^ He noted thereby the difficulty in determining this movement correctly either by calculation or observation:

Mr Daniel Bernoulli has also done something on this topic, and one finds something on it also in the work of Mr Maclaurin. I seem to recall that in reading Mr Newton the thing which appeared to me most difficult was the calculation of the movement of the apogee, which if I am not mistaken does not agree well with the observations. It is true that it is difficult to determine this movement correctly either by calculation or by observations. But I do no doubt that you have as usual enlarged upon this matter.’^41^

In this regard, Cramer had been able to draw on what he had learned about Newton’s results from his Genevan friend Calandrini. At the same time, however, he was also in contact with Euler and d’Alembert, both of whom were also working on this and related topics.

Meanwhile, Clairaut recognized that he needed to establish a stronger claim to the revisions he was proposing to Newtonian theory, and therefore indicated to Cramer later the same year that he intended to put something on the topic in the *Mémoires* of the Académie. With this goal in view, he was keen to know precisely what Calandrini had done, and if there was something of his already in print that Cramer could send him.^42^ Staking his own claim was important to Clairaut especially since, as he complained, other commitments had prevented him from producing an independent publication of his own on the theory of the Moon up to then.^43^

At this juncture in their correspondence, Cramer informs Clairaut that Calandrini had at first forbidden him to send anything, reportedly telling him, Cramer, that what he had written was not worth sending.^44^ Only after much persuasion had Calandrini eventually given way and permitted Cramer after all to enclose a sample of his work. As Clairaut would see, Cramer suggests to his French correspondent, it was not in any way a question of false modesty on Calandrini’s part, but that quite simply the fact that the results of his investigations agreed with those of Clairaut, ‘or at least nearly so, since he is satisfied with an approximation’.^45^

True to his word, Clairaut produced his first *Mémoire* for the Académie on the theory of the Moon in July of the same year, and he immediately sent a copy to Cramer in Geneva. Upon its arrival, Cramer duly took it to Calandrini, the latter being, as he wrote to Clairaut, ‘more conversant in these matters’ and they also being ‘quite fresh in his mind, because of his work on Newton’.^46^ This was the first tacit mention of the Genevan edition of the *Principia* in the two men’s exchanges up to this point, and indeed it was sufficiently vague as to remain hidden. Moreover, though no doubt unknown to Clairaut at the time, Calandrini had been the author of a long annotation on the Moon’s apogee appended to the Geneva edition.^47^ A further communication from Calandrini was duly enclosed in Cramer’s next letter to Clairaut, and in this manner the debate between Clairaut and Calandrini continued with a high degree of civility—as both sides agreed—over the next 5 years.^48^

As the facilitator of this debate, Cramer was therefore well acquainted with the ongoing ostensibly private discussion on the theory of the Moon between Calandrini and Clairaut when he heard the French mathematician’s presentation at the Paris Académie in November 1747. What disturbed him was Clairaut’s public suggestion that he had been the first to discover a fault in Newton’s theory—although Clairaut did concede on that occasion that Euler and d’Alembert had also reached similar conclusions to his own.

## 6. The aftermath of Clairaut’s 1747 *Mémoire*

Roused by what he considered to be Clairaut’s false arrogation of glory in the *Mémoire* he presented at the Paris Académie in November 1747, Cramer immediately drew up a detailed report on the meeting and sent it to his Genevan friend, the mathematician and natural philosopher Jean Jallabert (1712−1768) 2 days later.^49^ His intention in doing so was to galvanize their other friends in Geneva into providing support for Calandrini’s claim to priority. It is a measure of the strength of the Genevan Republic’s small but self-assured mathematical community that this is precisely what happened. Already by the end of December 1747, De la Rive sent a letter to Cramer in Paris, re-affirming Calandrini’s claim and supposing at the same time that what he wrote would be no different from what other common friends, Jacob Vernet (1698−1789), Jallabert and Calandrini himself, had to say:

M. Clairaut has set out in his *Mémoire* that he was the first who discovered the small gap which the movement of the apogee leaves in the system of Newton, in so far it diverges by half from what it should be according to the principle of attraction or of gravitation. You know that our friend M. Calandrini observed the same error when he calculated the movements of the moon, and that he made a note about this in his Newton. Even if this is nothing but a tiny weakness in his system, which likely results from some error in calculation or from some slightly inexact assumption, or from the fact that one does not yet grasp perfectly enough the orbit described by the moon, or some other unknown cause, it appears to me impossible that the principle of attraction could explain thousands of the most complicated phenomena, and this satisfactorily, if it were not the true system of the world.^50^

In concluding his letter, De la Rive left no doubt as to where he stood regarding the conclusions Clairaut had sought to draw:

Nevertheless, certain words that have been put in the Gazettes in this regard suddenly allow those to triumph who, being little or not philosophical, are happy to claim that there is nothing certain and settled in physics and also permits them to speak as if M. Clairaut had overturned the system of Newton: precisely he who, as you have indicated to M. Jalabert, recognizes that he has found in it all the calculations correct and corresponding well with the phenomena, with the exception of this minor circumstance.^51^

In the same month, Calandrini himself wrote to Cramer, recalling that in comments on Newton’s theory of the Moon he had published in the Genevan edition of the *Principia*, he had drawn attention to the deficits in that theory, while at the same time emphasizing that there was no need to modify the law of attraction.^52^ Some two months later, on 28 February 1748, De la Rive reported to Cramer that Calandrini had spoken about Clairaut’s *Mémoire* to the learned Saturday Society in Geneva soon after he had received it, and that he had described, at the same time, the response he had drawn up and sent to Cramer. Clearly, that response was the letter of Calandrini referred to by De la Rive in October as part of a four-pronged refutation of Clairaut.^53^ Incidentally, De la Rive expressed his perplexity at Clairaut’s desire to look for another law than that of the square of distances in order to explain apsidal precession.^54^

**Figure 1.**
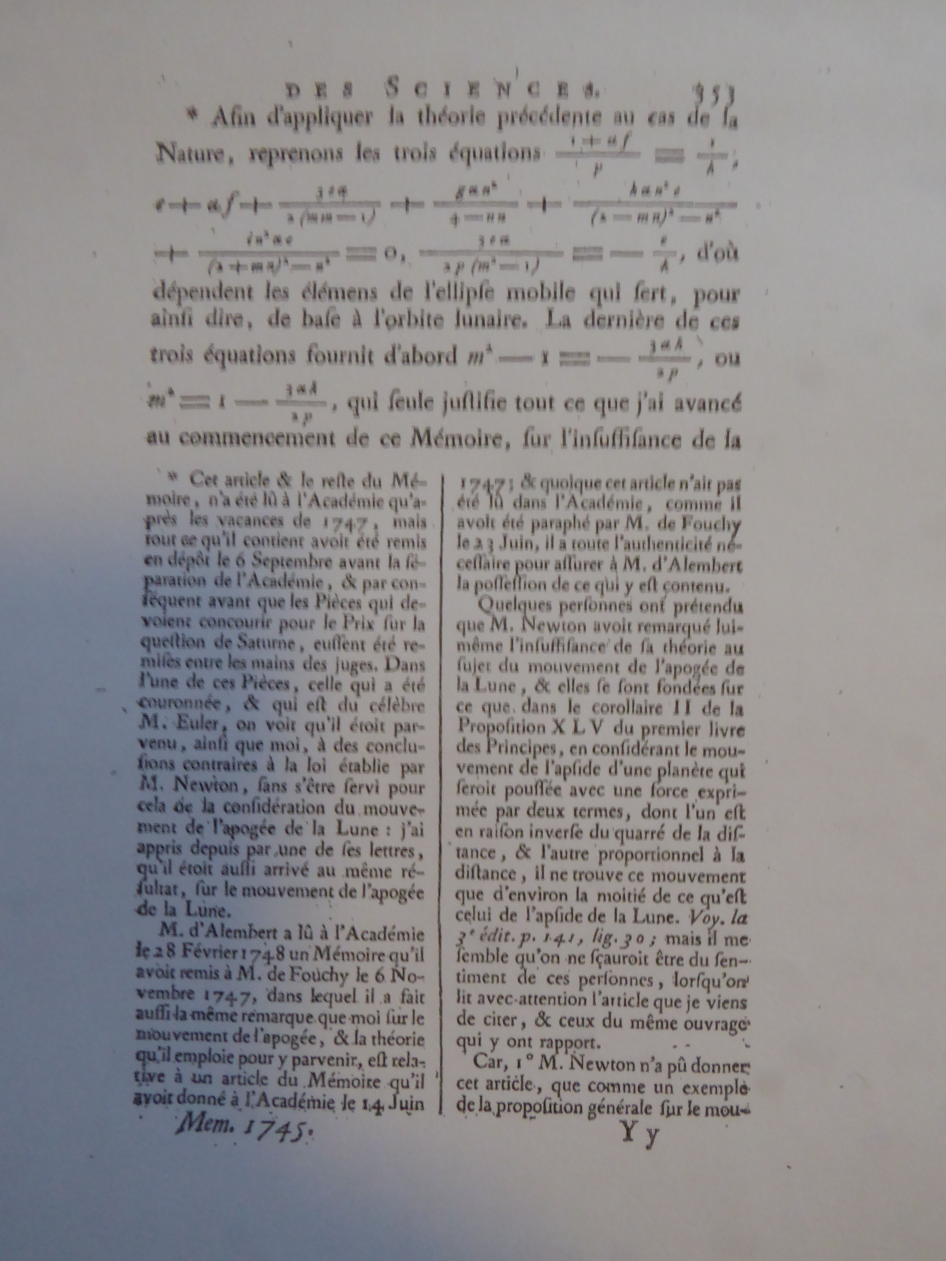
Clairaut, Du système du Monde dans les principes de la gravitation universelle. *Histoire de l’Académie Royale des Sciences*, vol. 1745. Paris 1749, p. 353.

As already indicated, Clairaut was probably not aware before late 1747, when he delivered his *Mémoire* at the Paris Academy, of the long note on the apogee of the moon’s orbit that Le Seur and Jacquier had included in the third volume of their edition of the *Principia*. For his part, Cramer only referred in passing to Calandrini’s work on Newton, while the edition itself was never mentioned explicitly. In fact, the first time Clairaut talked about the Geneva edition in print was in 1750, when the 1747 *Mémoire*, with substantial additions resulting from his subsequent investigations, was published by the Académie (figure 1). That *Mémoire* now included a note by Clairaut quoting passages from the long commentary on *prop*. 35, *probl*. 16 in the third volume of the Geneva edition that had clearly been written by the Genevan mathematician, although Calandrini’s authorship was not explicitly indicated.^55^ It is very likely, however, that Cramer drew Clairaut’s attention to the edition while he was in Paris and, around the same time, showed him the letters that had arrived from De la Rive, Jallabert and Calandrini. Not for the first time, an occasion had indirectly presented itself in which Genevan mathematicians could confidently seek to assert the significance of their own scientific achievements.

Unfortunately, Clairaut was not one to accept being corrected publicly. Almost mockingly, he subsequently sought to undermine Calandrini’s conviction that nothing in the *Principia* itself served to demonstrate the insufficiency of the inverse square law, and that only the method employed to ascertain the movement of the apogee was defective, because Newton did not consider the eccentricity of the lunar orbit, but instead regarded it as nearly circular. All those who believed Calandrini had recognized the true source of the problem in Newton’s theory before he, Clairaut, had done so were, he suggested, themselves in error.^56^

## 7. Clairaut and priority in discovery

In the published version of the 1747 *Mémoire*, Clairaut contended that of all the inequalities that affected the lunar orbit what was most in need of investigation was the movement of the apogee, this being also what Newton had treated of most obscurely.^57^ However, after Clairaut had applied to Newton’s calculations all the accuracy they demanded he found that they yielded a movement of the apogee that was at least twice as slow as what observations revealed: ‘that is to say, that the period of the apogee that followed from the reciprocal attraction proportional to the squares of the distances would be around 18 years, whereas in reality it is a little under 9’.^58^ Newton himself admitted that he did not think his calculations to be exact enough.^59^ For Clairaut at this time, the consequence was serious. A theory that could not deliver the movement of the apogee or whose results could not be blamed on observational errors was ‘from then on condemned without appeal’.^60^

Clairaut also communicated his results within his correspondence network to those who were working on similar topics. Thus, in two letters he sent to Euler in September 1747, around the time the Parisian Academy went into recess, he remarked that in respect of the Moon’s apogee Newton’s model delivered a value that was barely the half of what is observed in nature, and that this appeared to him to provide ‘the most complete proof of the insufficiency of the inverse-square law’.^61^ It was true, he admitted, that by adding some other term the theory could be made to agree enough with the phenomena, but this term would need to be such that it made a considerable difference to celestial bodies such as the Moon and the Earth attracting at a small distance, while almost vanishing for bodies such as the Earth and the Sun attracting at a great distance. The term Clairaut subsequently ascertained was published in a long additional section added to the 1747 *Mémoire*, which he fashioned as a new law.^62^

Clairaut sent sealed accounts of his new discovery—without the underlying workings—to among others the presidents of the Royal Society, Martin Folkes (1690−1754), and the Paris Academy, Armand Louis d’Aiguillon (1683−1750). In doing so, he used an established procedure to stake his claim to priority, while allowing himself more time to check and reassess his calculations before going public. The recipients of these hidden communications were requested not to open them until they received permission to do so from the author. The employment of this strategy was all the more pertinent given the number of high-ranking scholars who were working on aspects of the theory of the Moon at the time.

Sometime later in 1748, however, Clairaut came to realize that while there was indeed a gap in Newton’s observational calculations the inverse-square law was otherwise correct. This was the view that had been advocated all along by Calandrini. Hence, Clairaut found that he had no alternative than to retract his earlier revised theory, and he proceeded to do this in a paper he presented to the Académie des Sciences on 17 May 1749.^63^ A few weeks later, on 3 June 1749, he sent a somewhat disingenuous account of this retraction to Cramer from Paris. He begins by reminding the Genevan mathematician that some six months earlier he had sent copies of his *Mémoire* to England and France as well as to his friends in Basel. Only after doing so had he happened upon an idea concerning the apogee of the Moon that was so complicated he could not imagine anyone having thought of it before. He then proceeds:

Since it was very important for me not to let anyone forestall me in this matter, I sent a sealed parcel to London which contained my new result, and I urged Mr Foulkes not to open it until I asked him to do so, and I took the same precaution here at the Academy. My intention thereby was to avoid being anticipated by someone who wanted to boast that he had corrected me and to give myself more time before announcing my retraction so that I could complete the calculations that led me to it.^64^

It was, Clairaut explains further, extremely fortunate that his public announcement of retraction occurred at the same time as the printed version of his *Mémoire* left the press, this saving him from embarrassment. But what was the new idea he had happened upon? Clairaut sets it out in just a few words:

[…] after having considered the question from a point of view that had not been considered by anyone before I have arrived at finding the true movement of the apogee without employing any other force than that which acts by the inverse of the square of the distance.^65^

The problem had been essentially methodological in nature. Clairaut had succeeded in explaining one of the irregularities in Newton’s theory by noticing that some higher-order perturbative terms that had up to then been ignored were actually important, leaving the inverse square law unchallenged.^66^ Half-heartedly, he concedes that Calandrini could be considered the victor, insofar as Newton’s law of attraction remains intact, but at the same time suggests that all those who understand the matter well would recognize that the eccentricity on which the Genevan mathematician places so much weight was a long way from providing him with what he wanted, even if it be considered infinitely small. Far from truly acknowledging victory to Calandrini, Clairaut rounds off with a damning verdict: ‘The errors which I begrudge in his solution are such that not a single geometer would consider the problem to be solved, nor the theory of the Moon to have been found.’^67^

For the time being, he remained silent on the details of his new results, but in the definitive version of his *Mémoire* that was published under the auspices of the Russian Academy of Sciences at St Petersburg in 1752, he indicated the path along which he now intended to proceed.^68^ He was however unwilling in that august scientific body to be entirely open about the earlier course of the discussion on the theory of the Moon, and in particular his role in it, by providing a short account in which his identity is obscured:

Despite the large number of fine investigations on the cause of the irregularities of the Moon which have appeared in recent times, it is necessary to agree that the theory of gravitation, upon which all these investigations are founded, has not yet received all the insight it should get in such an important topic. One of the most important things it encompasses, the revolution of the apogee of the Moon, has been the cause of many diligent discussions and provided the pretext for proposing additions to the general law of forces. In truth, one of the mathematicians who had taken recourse to this expedient has retracted it and announced that he has found the means of deriving from his theory the correct movement of the apogee without employing any other force than that which follows from the inverse proportion of the square of distances.^69^

Euler’s response was understandably euphoric. Writing to Clairaut from Berlin, he congratulated the French mathematician on his happy discovery. It was, in his opinion the most important discovery in the theory of astronomy, without which it would be impossible to achieve a sound understanding of the mutual perturbations that the planets bring about in the course of their movement:

‘For it is very certain that it is only since this discovery that one can consider the law of attraction reciprocally proportional to the squares of the distances as solidly established; and on this depends the entire theory of astronomy.’^70^

## 8. Calandrini, Cramer and the Geneva edition

With the two principal editors Le Seur and Jacquier based at the Convent of Trinità dei Monti in Rome, it was Calandrini who was tasked with giving the Geneva edition its final form and seeing it through the press.^71^ Unfortunately, we have no direct evidence on this collaboration, because none of the correspondence between the two Minim friars and Calandrini has survived. No doubt they sent their draft comments to Geneva on a regular basis with all the vagaries that postal communication at that time presented. The arrangement evidently worked amicably, although Calandrini conceived his editorial task rather differently to that of his colleagues in Rome. While Le Seur and Jacquier sought primarily to elucidate Newton’s often impenetrably dense prose, Calandrini aimed at closing visible gaps in the scientific argument, thereby adopting a far more critical stance. He also introduced arguments and material from other authors reflecting the discussion that had taken place during the more than 50 years since the *Principia* was first published.

The two priests go some way to acknowledge this difference in approach in notes they included at various places in the work’s three volumes. As they point out at the beginning of the first volume, Calandrini took on a considerable number of practical tasks such as engraving the diagrams and arranging their position in the text or correcting typographical errors. He also composed a large account on the elements of conic sections and other things that did not appear to be sufficiently explained in the original work.^72^ No less importantly, it was Calandrini, too, who assisted in the crucial task of obtaining funds for the edition to be printed.

Getting the Geneva edition into good shape required a consummate mathematician such as Calandrini clearly was.^73^ He corrected certain mistakes made by Le Seur and Jacquier, explicated various passages in the *Principia* they had failed to rescue from obscurity, and filled several gaps they had passed over. The division of labour was also made apparent for the attentive reader: all the notes indicated by an asterisk in the final work are down to the Genevan mathematician, but there are many more besides not indicated in this way.^74^ Their range is enormous, stretching from the inclusion of a veritable tract on conic sections in the first volume to a refutation of the vortex theory upheld by Johann I Bernoulli in the second. The third volume, as has already been mentioned, contains a long piece by Calandrini on the movement of the Moon. De la Rive’s no doubt half-hearted attribution of the Geneva edition to his friend and colleague did perhaps have a certain justification.

**Figure 2.**
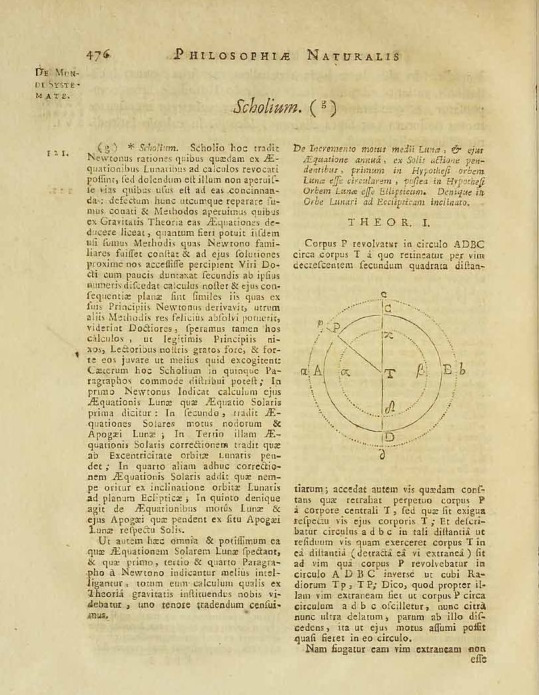
Calendrini’s annotation on *prop*. 35; Newton, *Principia*, Geneva 1742, III, 1, p. 476.

By contrast, Cramer’s contribution to the edition is small but nevertheless remarkable, because it consists in a note appended to Newton’s proposition 47, Book II, in volume two which Le Seur and Jacquier specially commissioned from him and printed in full (figure 2).^75^ We know that Jacquier met with Cramer a number of times in Geneva when he travelled to or from his native France, and that the two men held each other in high esteem.^76^ But what was the reason for Cramer’s note? Le Seur and Jacquier were convinced that Newton’s theory of sound was essentially correct, but they were unhappy with its derivation and sought to supplement the original text with a new proof. Indeed, as Cramer explains in his note, it is not difficult from the proof of Newton’s theory to draw other conclusions from the one he does. Interestingly, the reviewer in the *Neue Zeitungen* makes a point of drawing the attention of the journal’s readers to Cramer’s contribution:

Gabriel Cramer has shown that Newton’s proof is not conclusive, by deriving a completely different proposition along precisely the same lines. The two priests have inserted Cramer’s argument word by word. They also believe that Newton’s assertion is correct, but that it is insufficiently demonstrated by him, and therefore seek to provide a new proof.^77^

To the learned public, the Genevan mathematicians Calandrini and Cramer were thus presented in the *Neue Zeitungen* and elsewhere as having made important interventions to correct identified defects in Newton’s original work.

## 9. Cramer between Geneva and Basel

Although Cramer was not directly involved in editing the Geneva edition of the *Principia*, he was sufficiently close to those who actually were to be seen by contemporaries as something of an intermediary. This was particularly true for members of the Bernoulli family, to which as we have seen, he had close ties resulting from the time he spent in Basel at the beginning of his scientific career. Thus, in November 1743, Daniel Bernoulli, who exchanged many letters with Cramer although the two men never met, wrote from his native Basel to the Genevan mathematician to express his surprise, if not to say dismay, on account of a rumour he had recently heard. Some 3 years earlier, he had submitted a contribution to a prize question of the Paris Académie des Sciences to find and elucidate the true cause of the flux and reflux of the sea. It now had transpired that the three treatises awarded prizes, one of which was his own, had simply been reprinted in the third volume of the Genevan edition without any prior warning having been given to their authors. The editors of the edition had therefore done him a disservice. As Daniel politely points out to Cramer, he would have liked to have been advised by them of their intention beforehand, because he could then have made numerous additions to his piece ‘in order to show the remarkable agreement there is between my theoretical ideas and experiments or observations, of which I was not aware beforehand’.^78^

The following year, Daniel Bernoulli informed Euler, whom he had worked alongside for 7 years at the Academy in St Petersburg, that his *Preisschrift* together with both that of Colin Maclaurin (1698−1746) and his own contribution had been printed in full by Jacquier and Le Seur. It was the inclusion of these tracts that had required the third volume to be split into two parts.^79^ This time, however, Daniel was full of praise for the editors of the Geneva edition. ‘In Geneva, Newton’s *Principia* has been reprinted with many good annotations, in which you are very often quoted. Your piece on the tides has also been reprinted in full word for word along with that of M. Maclaurin and my own’, he writes.^80^ As elsewhere in their correspondence, the tone of Bernoulli’s remarks to Euler was highly reverential, and he was no doubt concerned not to pre-empt Euler’s own judgement on the work of Calandrini, Jacquier and Le Seur.

While Euler on all occasions recorded in his correspondence speaks approvingly of the Geneva edition, things are rather different with members of the Bernoulli family. Some six months before Daniel’s letter criticizing the unsanctioned reprinting of his tract on the theory of the tides, his brother, Johann II Bernoulli wrote to Cramer from Basel to put to him the rather malevolent question, which had earlier been raised by a friend as to the purpose and value of the Geneva edition. One suspects that Johann II had no misgivings about relaying this question, which he slips in innocently after first conveying to him the friendliest greetings of his father, that is to say, Johann I Bernoulli, Cramer’s former teacher and a sworn opponent of Newton:

These are also the sentiments of my father, who has asked me to assure you at the same time of his entire friendship. A friend has requested that I ask you, Sir, what are Newton’s Principles of Natural Philosophy with commentaries by fathers Le Seur and Jacquier which have been printed in Geneva, and if this book is worth buying.^81^

## 10. The Bernoulli and the Geneva edition

There is no reason to think that Cramer had the slightest doubt as to the value of the work produced by Le Seur and Jacquier. Some 2 years later, he had no difficulty in suggesting to Johann II Bernoulli that his ageing father might be prepared to accept a copy of the Genevan edition of the *Principia* in exchange for his copy of the third London edition on which it is based. Evidently, it was hard to get hold of the 1726 edition on the Continent. The contemporary interest is understandable, since it was known that in this edition Newton sought to deliver a clearer explanation of the gravitational components of the Earth and the Sun in determining the precise nature of the lunar orbit.^82^ The circumstances of Cramer’s letter to Johann II are enlightening not just because of what it tells us about the accessibility of the third edition, but also because it reveals that Cramer was in contact with Ulrich Baron von Thun (1707−1778), Court Councillor to the court of Saxe-Gotha-Altenburg, before he travelled on his fateful journey to Paris in 1747. Furthermore, it provides us with a previously unknown detail of how the Genevan edition was produced in the first place.

In 1743, von Thun had been appointed by Duchess Louise Dorothea (1710−1767) as instructor to her son, the hereditary prince Frederick-Louis. During a long sojourn in Geneva from August 1744 to April 1747, enforced by the duchess because of the occupation of German lands during the ongoing War of Austrian Succession,^83^ he had become acquainted with Cramer and subsequently engaged him as the boy’s mathematics tutor in Paris.^84^ Surviving correspondence between von Thun and Louise Dorothea reveals that Cramer did not have an easy time. Despite having been initially praised to her as a ‘perle’, Cramer was by now considered by von Thun as not being worth his expense and as being easily replaceable.^85^ The contrast between highly admired savant and downtrodden tutor could scarcely be greater.

As Cramer relates in his letter to Bernoulli, Baron von Thun, whom he describes as ‘a personal friend of Maupertuis’, had been looking everywhere for a copy of the 1726 edition, yet not for himself. Rather, he was seeking to obtain it for Gabrielle-Émilie, Marquise du Châtelet (1706−1749) who was at this time working with Clairaut on the French translation of the *Principia*.^86^ Maupertuis’s own search had been unsuccessful, and so Cramer, who held Mme. Du Châtelet in high esteem, having been introduced to her by Jacquier, was keen to help her out. At the same time, he was no doubt eager to please his former employer.^87^

Cramer concedes that he himself had possessed a copy, but that he had given it to Barrillot to serve as printer’s copy in preparing the Genevan edition. He makes no mention of Calandrini, but instead seeks to prevail upon Johann II Bernoulli in a manner that comes across as rather desperate and heavy-handed. Apart from the possibility of a straight swap, Cramer offers to procure another copy of the 1726 edition from London—something which would of course have taken time—or simply to pay for the one Johann II’s father owned. Clearly, there was no time to lose in enabling Mme. Du Châtelet to complete her work. Cramer seemed willing to help her even at the cost of alienating his friends in Basel:

Mr baron de Thun, a special friend of Mr de Maupertuis, has looked everywhere for the final London edition of Newton’s *Principia*. It is for marchioness du Chatelat who has dealings with it. We are unable to find it here. I had a copy, but I gave it to Barillot for the edition he has produced of this work with the commentary of the priests Leseur & Jacquier. I have been thinking, my dear sir, that it would not be impossible to find a copy amongst your good selves. And if I am not terribly mistaken, sir, your father must have one. We should be very obliged if he would agree to do this. I can after all easily get another from London; at least if he does not prefer that one pays for the value or if he did not want to receive in exchange a copy of the Geneva edition with the commentary. If you, sir, or some other owner of this edition might indeed be willing to undertake this kindness, kindly have that of taking care to send it to Paris to madam du Chatelet by the most suitable and shortest route.^88^

This request was not only heavy-handed, but also disingenuous. Cramer had good reasons for believing that Johann II’s father had a copy of the 1726 edition, because he had in fact given him one as a present. No doubt somewhat bemused by the nature of Cramer’s request, Johann II nonetheless duly expedited his father’s copy to Paris by his preferred means, while Baron von Thun undertook to procure a replacement copy for Johann I Bernoulli from London. As for the offer of a copy of the Geneva edition, Johann II politely declined, revealing that his father possessed one already.^89^

Mme. Du Châtelet later, in September the following year, suggested to her friend Johann II Bernoulli that without his help in procuring a copy of the third edition of the *Principia* her task of preparing the French translation of the work would scarcely have been possible. But that was not all. Since that task also involved her providing a commentary, Du Châtelet also wished to consult the Geneva edition as her work proceeded, especially the second part of volume 3 devoted to Newton’s theory of the Moon. In fact, she was most in need of the two parts of the third volume of what she calls ‘Jacquier’s Newton’, on account of her close friendship with the younger of the two Minim priests. Her request for the third volume was no doubt on account of Calandrini’s substantial commentaries that this volume contained. Thus, while thanking Bernoulli once more for his assistance in the past she now stressed to him her urgent need for the third volume. Presumably because up to this time she had not seen any volumes of the Geneva edition, she mistakenly assumed that Bousquet, the publisher of Cramer’s editions of the works of Johann I and Jacob Bernoulli, would also be the printer of the annotated edition of the *Principia*:

You have already had the kindness to procure for me a 1726 edition of Newton, which I could not have translated without you, for want of having the good edition. I must again take recourse to you to beg you to obtain a Jacquier for me. One should ask M. Bousquet in Lausanne. […] I would be bound, Sir, with the greatest obligation to you, because I completely lack these [sc. volumes] and have an extreme need of them.^90^

Others were likewise able to claim credit for the assistance they had provided. Baron von Thun, whose initial efforts to find the third edition of the *Principia* had proved so fraught, was able to inform his patron Duchess Louise Dorothea with evident satisfaction that he had obtained for Mme. Du Châtelet a rare impression of the *Principia* as an aid to her translation.^91^

The revelation that Cramer supplied Barrillot with his copy of the 1726 *Principia* to aid production of the Geneva edition should not surprise us, given his excellent connections to publishers in his native city. Quite possibly, that copy would have contained some of the corrections and annotations he, and possibly also Calandrini, had collected, but we shall never know for certain how the work was finally composed in the printerʼs workshop. Given Cramerʼs experience, it is very likely that he would have corrected the proofs, but in this respect, too, no details have survived.

It was as a potential corrector that Cramerʼs acquaintanceship with Euler came about in 1743. At the request of the publisher Marc-Michel Bousquet, Euler wrote to Cramer asking if he would correct the proofs of his small tract on the isoperimetric problem.^92^ Euler also gave Cramer the opportunity of writing a preface to the tract, but Daniel Bernoulli volunteered to do this instead.^93^ The tract eventually appeared, however, without a preface. Over a year later, Bousquet approached Cramer again and with a similar request, this time to correct the proofs of Euler’s *Introductio in analysin infinitorum*.^94^ On this occasion, Cramer refused, citing both the large amount of other business he needed to attend to, and the fact that the publication he was working on himself covered the same material as the second part of Eulerʼs work.^95^ Clearly, reasons of intellectual propriety precluded his correcting the proofs of Eulerʼs work, but the delay incurred by his preoccupation with other matters came at considerable cost. It was a good 2 years before he was able to publish his own contribution to the topic, the *Introduction à l’analyse de lignes courbes algébriques*, which because of its late appearance immediately fell under the shadow of what Euler had brought out before. When, in August 1650, Cramer sent Nicolaus Bernoulli a copy of the printed pages of his work seeking his assessment of it, he at the same time, voiced his frustration over the delays in its publication: ‘It is truly a long time since it should have appeared, had not various inconveniences, both imposed on my part and by various booksellers, delayed publication right up to today.’^96^

## 11. Cramer as editor and author

As revealed by the contemporary letter to Nicolaus Bernoulli, there is strong evidence that Cramer had completed a large part of his *Introduction* by the time Bousquet approached him with his request. Despite the averred similarity of aims, there is practically no methodological overlap in the two works, making the delay to the appearance of Cramerʼs *Introduction* all the more tragic. It is a measure of the Genevan mathematicianʼs intellectual generosity that when his work eventually came out he expressed his regret at not having seen Euler’s work sooner than he did:

I could have drawn great use from the *Introduction à l’analyse des infiniment petits* of M. Euler if that volume had been known to me sooner. The object being almost the same as my own, it is not surprising that we have often met in our conclusions. Nevertheless, the difference in methods is as big as can be when one writes on the same topic. Which I do not say because I prefer the route I have taken to that of M. Euler, but only to inform the reader of this difference.^97^

Cramer’s work appeared under the imprint of his cousins, Gabriel and Philibert Cramer, together with Claude Philibert, who could also count Voltaire and Étienne de Condillac (1714−1780) among their clients. The Genevan mathematician himself conducted an extensive correspondence with both of these illustrious men of letters.^98^ Despite Cramer’s *Introduction* having been eclipsed by Euler’s *Introductio*, it could nonetheless claim considerable scientific importance in its own right. Thus, it presented for the first time a considerable number of curves that the author had discovered, along with a complete classification of algebraic curves based on the number, species and position of their infinite branches.^99^ Cramer acknowledged his indebtedness to Newton who, in 1704, as an appendix to the *Opticks*, published a classification of cubic curves under the title *Enumeratio linearum tertii ordini*s.^100^ Before Newton only a small number of such curves had been known. Cramer praises the classificatory model established by Newton but deplores the fact the English mathematician had seen fit to publish his discoveries without at the same time including their demonstrations. As Cramer puts it, that Newton had preferred the pleasure of being admired rather than of providing instruction.^101^ In fact, this is only partly true.^102^ Furthermore, the author also announced a general rule for solving simultaneous linear equations that today bears the name ‘Cramer’s rule’.^103^ Although Leibniz had succeeded in deriving this rule before him in the context of his studies on the calculus of determinants, his work on this topic was unknown at the time and indeed has only recently been published from his extensive mathematical *Nachlass*.^104^

Some of the editions whose preparation took up so much of Cramer’s time have already been mentioned. But it should not be forgotten that alongside the works and letters of Christian Wolff, Leibniz and Johann I and Jacob Bernoulli he also edited a three-volume collection of Newton’s *Opuscula* under Bousquet’s imprint in 1744. It is a further reflection of the tightly knit circle of mathematicians and publishers that existed in eighteenth-century Geneva that Cramer produced the Newton edition in collaboration with Jean Castillion, who had taken on the correction of Euler’s *Introductio* after Cramer had bowed out [54]. His collaboration with Castillion manifests another part of Cramerʼs scientific legacy. Both through his publications and his scientific contacts, the Genevan mathematician was ideally placed to mediate between the Leibnizian and the Newtonian camps at a time when scientific Europe was riven by philosophical division. In this respect, Cramer’s role could be compared favourably with that of Pierre Varignon (1654−1722), who could likewise claim to possess good ties on both side of that divide.

Following the conclusion of his long-lasting debate with Clairaut over the theory of the Moon, Calandrini exchanged his chair in philosophy at the Académie de Genève for a career in the political administration, initially becoming state councillor, but eventually rising to syndic, the highest post in the Republic. His move was in many ways befitting in view of the wealth and influence of the family from which he stemmed, but it was lamented in scientific circles. His successor on the philosophy chair was none other than Cramer, who despite the professional benefit to himself through the chair becoming vacant, regretted Calandrini’s departure from the Académie.^105^ In a letter to Clairaut, he poignantly describes Calandrini ‘as a man who has been lost to mathematics’, since his friend’s duties as councillor now occupied him entirely.^106^ At the same time, Cramer also points out that his friend had specifically asked him to say how pleased he was over Clairaut’s choice of words in his *Mémoire* of 1745.

Over the next 2 years, Cramer’s health declined and in consequence he moved to his wife’s native France, where he believed the change of climate would be beneficial to his health. Alas, the move proved to be of no avail: Cramer succumbed to illness at Bagnols-sur-Cèze in Languedoc on 4 January 1752. Not long afterwards, Daniel Bernoulli wrote to Jean Jallabert who had earlier succeeded Cramer on the Genevan mathematics chair to pass on the sad news:

I have lost an intimate friend. Your city and our Switzerland have lost one of its most precious ornaments, and all of Europe has lost a scholar of the first order, born to increase and perfect scientific knowledge […] He was not only a man of renown, but also a kind man of learning.^107^

## 12. Conclusion

There was a small but productive mathematical circle in Geneva that contributed substantially to the realization of the annotated edition of the *Principia* that emerged under the direction of Jacquier and Le Seur in Rome. Cramer and Calandrini, the key figures in the Genevan Republic, oversaw between them the financial side of the enterprise and its printing. They were ideally placed to do so given Cramer’s close family ties to the local book-trade and Calandrini’s political influence. Both men made important contributions to the commentaries, greatly facilitating access to Newton’s text, although numerically the number of notes supplied by Calandrini far outweighed those of his friend. There was an important difference in their scientific aspirations, too. Calandrini was evidently at ease with the local focus of his activity, first as teacher and later as politician. Despite his tremendous mathematical skill he did not seek fame. Cramer on the other hand slotted himself seamlessly into contemporary scientific discourse, and succeeded in achieving considerable recognition well beyond the borders of the Swiss Confederation and the Genevan Republic. An important stage in his scientific apprenticeship was the time he spent in Basel where he expanded his knowledge of contemporary work in mathematics under the tuition of Johann I Bernoulli and established a mutually beneficial friendship with Johann’s nephew Nicolaus. Through his travels to England, the Low Countries, and particularly France, he was able to establish his reputation in the Republic of Letters as a man of considerable talents in mathematics who could move at ease between the two rival camps that dominated the mathematical sciences for a large part of the century. His scientifically momentous sojourn in Paris in 1747 reveals, however, another side of scholarly life in the eighteenth century that is easily forgotten: without the support of a munificent patron even the most talented mathematicians sometimes had to earn their keep the hard way.

## Data Availability

This article has no additional data.

## Footnotes

^1See ^
[1], pp. 54–56.

^2^
Leonhard Euler to Christian Goldbach, 1 August 1741; see [2], pp. 167–169.

^3^
Christian Goldbach to Leonhard Euler, 19 August 1741; [2], pp. 169–170: ‘In den Zeitungen von Gelehrten Sachen habe ich unlängst gelesen, daß die beyden Mönchen so *Newtoni Principia Math* herausgegeben Eurer HochE. *Mechanicam* starck gebrauchet haben.’

^4See ^
[3]. On François Barrillot (1684–1748) and his son, Jacques Barrillot (1709–1750) see [4], p. 355.

^5^
*Neue Zeitungen von Gelehrten Sachen*, 17 July 1741, p. 505: ‘[...] Newtons Werk so deutlich zu machen, daß es auch von einem nur mittelmäßig in der Meßkunst geübten Leser kann verstanden werden.’ On the background and rationale of the Geneva Edition see [5], pp. 419–421.

^6^
Ibid., 506: ‘Sie erklären aber in ihren Anmerkungen nicht nur Newtons Sätze, sondern sie bringen auch andere Entdeckungen bey. Was in den Leipziger Actis, in den Geschichten der parisischen und petersburgischen Akademien, und in Herrn Hermanns Phronomie hieher gehörte, das haben sie angezeiget. Insbesondere ist Herrn Eulers Mechanik stark von ihnen gebraucht worden. Aus einigen Schriften des Herrn Poleni haben sie ebenfalls viel Vortheil gezogen. Sie haben aber das, was andere Mathematici entdecket, entweder selbst beygefüget [...] oder sie verweisen den Leser auf Schriften, wo er solche erkläret findet.’

^7^
On the contributions of Jacquier, Le Seur, and Calendrini in bringing about the publication, see [4], especially pp. 354–359; [5], pp. 421–423, [6], p. 37, [7], pp. 134–137.

^8^
On Cramer’s Genevan background see [8], pp. ix–x, 7–10; [9], p. 11.

^9See ^
[9], pp. 16–22; [10], pp. 4–5.

**^10See^ :**[8], pp. 8–9.

**^11See^ :**[9], p. 22; [4], p. 348; [7], p. 130.

^12^
Cramer to Calandrini, 24 May 1727, Geneva, Bibliothèque de Genève, Ms Fr. 2855, f. 35–36: ‘Je vais régulièrement tous les jours chés Mr. Jean Bernoulli, et presque aussi souvent chés son neveu Nicolas Bernoulli […]. Je raisonne principalement avec le premier sur le Calcul différentiel et integral. Il m’a communiqué ses leçons manuscrites qu’il avoit données au Marquis de l’Hospital.’ See also [11], pp. 155–156.

^13^
See [12], pp. 222–223.

^14^
Johann I Bernoulli to Daniel Leclerc, 13 May 1727, Universitätsbibliothek Basel, UBH L Ia 50, No. 7: ‘Je suis bien aise de connoitre personellement Mons. le Prof. Cramer; il me fait l’honneur de frequenter mes leçons sur la Geometrie des infinis, j’ai déja pû entrevoir qu’il a du savoir et du merite, tout jeune qu’il est; J’augure de Lui qu’il deviendra grand et excellent Mathematicien.’

^15^
Johann I Bernoulli to d’Ortous de Mairan, 28 September 1727, Bibliothèque de Genève, D.O. autogr. 4/22: ‘Le Porteur de cette lettre nommé M. Cramer est Professeur des Mathematiques à Genevé: c’est un Homme de grand merite et tres habile Mathematicien, quoique encore fort jeune; ayant obtenu de ses Souverains la permission de voyager il a fait son premier Sejour ici pendant environ 5 mois dans le dessein de profiter quelque chose de mes faibles lumieres […] Apres d’Angleterre il reviendra à Paris pour y rester quelque temps, son dessein étant sans doute de faire connoissance avec les Savants et de s’exercer dans les observations Astronomiques.’

**^16See^ :**[9], pp. 28, 32–34; [10], p. 5.

^17^
See [9], pp. 34–36; [4], pp. 348–349.

^18^
On these debates see for example [13].

^19^
Gabriel Cramer to James Jurin, 18 January 1729, *Royal Society*, Early Letters C2, No. 80; [14], p. 378.

**^20See^ :**[15]. Machin’s *The Laws of the Moon’s Motion according to Gravity* is found at the end of volume two.

^21^
Nicolaus Bernoulli to Gabriel Cramer, 2[3] March 1720, Universitätsbibliothek Basel, L 1a 21:1, f. 26r–28r: ‘Je profite de l’occasion, qui se presente par le depart de Mr. le Prof. Klinguenstirn pour Paris, pour repondre à l’honneur de vos dernieres lettres, et pour vous procurer en même tems le plaisir de faire connoissance avec ce savant Mathematicien, qui de son coté a une grande envie de connoitre votre personne, pour laquelle il a comme une haute estime fondée sur le portrait favorable et juste que mon Oncle et moi lui avons fait de vos belles qualités.’

^22^
Ibid.

**^23See^ :**[9], p. 37.

^24^
See [16], pp. 198–199; [8], pp. 69–80.

^25^
Cited in [17], pp.1–2: ‘Quelle est la cause de la figure elliptique des Orbites des Planètes, et pourquoy le grand axe de ces Ellipses change de position, ou ce qui revient au même, pourquoy leur Aphélie, ou leur Apogée répond successivement à differens points du Ciel?’

^26^
Ibid., Avertissement (unpaginated): ‘Les autres sont la Piece No. 3 dont la Devise est ‘Sicut tenebrae ejus, ita et lumen ejus’ […]’. See [11], p. 159. See also [10], p. 5, and [9], pp. 49–51.

^27^
Gabriel Cramer to Johann I Bernoulli, 7 April 1730 (minute); Bibliothèque de Genève, Ms Fr. 656, f. 16.

^28^
Calandrini had undertaken his Grand Tour from 1724 until 1727. See [9], p. 28.

**^29See^ :**[18], p. 94; [10], p. 7.

**^30See^ :**[19]. See [9], p. 79.

^31^
[20]. See [9], pp. 72–76. Johann Bernoulli wrote Cramer a letter of thanks, dated 1 May 1743; Bibliothèque de Genève, Ms Suppl. 384, f. 37r–38v.

^32^
[21]. See [9], pp. 76–77.

**^33See^ :**[22].

^34^
See [23], p. 368; [16], pp. 225–226; [24], p. 191.

^35^
Von Thun announced his intention to engage Cramer, describing him as a ‘perle’ in his letter to Duchess Louise Dorothea sent from Geneva on 20 January 1747, Thüringisches Staatsarchiv Gotha, Geheimes Archiv, E XIII a 26, f. 7r–7v; [25], p. 236–237.

**^36See^ :**[26], p. 335; [27], p. 577.

**^37See^ :**[28], p. 141: ‘Apsis lunae est duplo velocior circiter.’ On Newton’s treatment of lunar precession see [24], p. 186–193; [29], p. 190; [30], p. 101.

^38^
See [26], pp. 336–339; [31], p. 173; [9], p. 96.

^39^
See Gabriel Cramer to Alexis Clairaut, May 1744 (rough draft), Bibliothèque de Genève, Ms Fr. 657b, f. 9; [16], p. 218; [7], pp. 134–135; [18], p. 91.

^40^
See [32], pp. 21–22.

^41^
Gabriel Cramer to Alexis Clairaut, March 1744 (rough draft), Bibliothèque de Genève, Ms Fr. 657b, f. 8; [16], p. 216: ‘Mr Daniel Bernoulli a fait aussi quelque chose là dessus et on trouve quelque chose dans l’ouvrage de Mr Mac Laurin. Je crois me rappeler qu’en lisant Mr Newton, ce qui me parut le plus difficile c’étoit le calcul du Mouvement de l’apogée, qui ne s’accorde pas bien aux observations, si je ne me trompe. Il est vrai que ce mouvement là est difficile à bien determiner soit par le calcul soit par les observations. Mais ne doute pas que vous n’avés développé tout cela à votre ordinaire, c’est à dire à merveille.’

^42^
See Alexis Clairaut to Gabriel Cramer, 13 April 1744, Bibliothèque de Genève, Ms Suppl. 359, f. 5-6; [16], p. 218.

^43^
Ibid.

^44^
Gabriel Cramer to Alexis Clairaut, May 1744 (rough draft), Bibliothèque de Genève, Ms Fr. 657b, f. 9; [16], p. 218: ‘Sa modestie l’a fait d’abord se defendre de me rien remettre pour vous, disant que ce qu’il avoit composé ne valoit pas la peine de vous etre envoié.’

^45^
Ibid.: ‘[…] j’ose vous assurer le connaissant aussi particulièrment que je le fais, que ce n’est point chez lui une fausse modestie, et que ses recherches s’accordent avec vos recherches, du moins à peu près puisqu’il s’est contenté d’une approximation.’

^46^
Gabriel Cramer to Alexis Clairaut, August 1744 (minute), Bibliothèque de Genève, Ms Fr. 657b, f. 13; [16], p. 219: ‘Aussitôt que j’ai reçu votre lettre […] avec votre beau Mémoire, je le portai à Mr Calandrin plus versé que moi dans ces matières et qui les a encore toutes fraiches à l’esprit, à cause de son travail sur Newton.’

**^47See^ :**[3], III, 1, pp. 476–518; [4], pp. 358–359; [33], pp. 163–164.

^48^
For an overview of the correspondence between Clairaut and Calandrini see [34], pp. 13–19, and [9], pp. 79–82.

^49^
Gabriel Cramer to Jean Jallabert, 17 November 1747, Bibliothèque de Genève, Ms 242 SHAG, f. 68–70.

^50^
Amédée de la Rive to Gabriel Cramer, 29 December 1747, Bibliothèque de Genève, Ms Fr. 657a, f. 5v: ‘Monsieur Clairaut d’en il donné dans son mémoire Mr. le premier qui ait découvert le petit vide que le movement de l’apogée laisse dans le systeme de Newton, en ce qu’il ecarte de la moitié de ce qu’il devroit être par le principe de l’attraction ou de la gravitation. Vous saves que notre Ami Mr. Calandrini avoit observe la même erreur quand il calculoit les mouvements de la lune, et qu’il en a fait une note dans son Newton. Quoy que ce ne soit la qu’un foible bien leger dans ce systême qui doit procéder de quelque erreur de calcul ou de quelque supposition peu exacte, ou de ce connoit pas encore assés parfaitement l’orbite que la Lune décrit, ou de quelque autre cause inconnue, puis qu’il me paroit impossible que le principe de attraction explique des milliers de phaenomenes des plus compliqués, et cela tres heureusement, sans que ce soit le veritable systeme du monde.’

^51^
Ibid.: ‘cependant quelques mots qu’on en a mis dans les gazettes font triumpher tous coup qui, peu ou point philosophes, se plaisent a dire qu’il n’y a rien de certain et de fixe en Physique, et les font parler comme si Mr. Clairaut avoit renversé le systeme de Newton, luy qui, comme vous le marquiés a Mr. Jalabert, reconnoit qu’il en a trouvé tous les calculs justes et bien répondants aus Phénomenes, à l’exception de cette légère circonstance.’

^52^
Jean-Louis Calandrini to Gabriel Cramer, 12 December 1747; British Library Add. MS 23899, f. 52–53. Calandrini’s comments (or rather tract) on *Principia* proposition 35, problem 16, Book III, are [3], III, 2, pp. 476–518.

^53^
Amédée de la Rive to Gabriel Cramer, 28 February 1748, Bibliothèque de Genève, Ms Fr. 657a, f. 7v.

^54^
Ibid.: ‘Je ne comprends pas non plus comment Mr. Clairaut a pu regarder comme petite distance, en fait d’attraction ou de gravitation celle de la lune a la terre, et chercher une autre loy que celle du quarré des distances pour expliquer le movement de l’apogée.’

^55^
[3], III, 2, pp. 476–518.

^56^
[26], p. 355: ‘ce qui a fait croire […] que M. Calendrin avoir reconnu avant moi le défaut de la loi d’attraction employée par M. Newton; mais il est aisé de voir par la suite du même article de M. Calendrin, qu’il n’est pas de mon avis sur cette matière, & qu’il croit seulement que la méthode employée par M. Newton, pour découvrir le mouvement de l’apogée, est défectueuse en ce point, qu’il ne considère pas l’excentricité de la Lune.’ See also Alexis Clairaut to Gabriel Cramer, 3 June 1749, Bibliothèque de Genève, Ms Suppl. 384, f. 140–141; [16], pp. 224–226; [7], p. 135; [4], pp. 367–368.

**^57See^ :**[26], p. 335.

^58^
Ibid., p. 336: ‘Après avoir mis à ce calcul toute l’exactitude qu’il demandoit, j’ai été bien étonné de trouver qu’il rendoit le mouvement de l’apogée au moins deux fois plus lent que celui qu’il a par les observations, c’est à dire que la période de l’apogée qui suivroit de l’attraction réciproquement proportionelle aux quarrés des distances, seroit d’environ 18 ans, au lieu d’un peu moins de 9 qu’elle est réellement.’

**^59See^ :**[35], p. 463: ‘Sed neque computationem nostrum satis accuratam esse putamus.’

**^60See^ :**[26], p. 336: ‘[...] elle seroit dès-lors condamnée sans appel’.

^61^
Alexis Clairaut to Leonhard Euler, 3 September 1747, [36], pp. 169–171; Alexis Clairaut to Leonhard Euler, 11 September 1747, [36], pp. 173–174: ‘Et ne le trouvant guères que la moitié de ce qu’il est dans la Nature cela m’a paru fournir la preuve la plus complete de l’insuffisance de la Loy du quarré.’ See [37], p. 28.

**^62See^ :**[26], pp. 353–364.

**^63See^ :**[31], p. 140, pp. 173–176.

^64^
Alexis Clairaut to Gabriel Cramer, 3 June 1749, Bibliothèque de Genève, Ms Suppl. 384, f,140–141; [16], p. 224: ‘Comme it etoit fort important pour moi de ne me pas laisser prevenir par personne sur une chose de cette nature, j’ai envoyé à Londres un paquet cacheté qui contenoit mon nouveau resultat en priant Mr. Folkes de ne le faire ouvrir que lorsque je le lui manderois et j’ai usé de cette même precaution ici a l’Academie. Mon intention etoit par ce moyen d’eviter d’etre relevé par personne qui put se vanter de m’avoir redressé et d’attendre a lacher moi même ma retraction que j’eussse achevé entierement le calcul qui m’y avoit conduit.’ See also [27], p. 578.

^65^
Ibid: ‘[…] j’annonce qu’apres avoir consideré la question sous un point de vue qui n’a pas encore eté envisage de personne je suis arrive a trouver le vray mouvement d’apogée sans employer d’autre force que celle qui agit en raison renversée du quarré de la distance.’

**^66See^ :**[24], pp. 191–192.

^67^
Alexis Clairaut to Gabriel Cramer, 3 June 1749, Bibliothèque de Genève, Ms Suppl. 384, f. 140–141; [16], p. 226: ‘Les erreurs que j’ai reprochées à sa solution sonte telles qu’aucun géomètre n’aurait pu regarder le problème comme résolu ni la théorie de la Lune comme trouvée.’

**^68See^ :**[38], p. 92.

^69^
Ibid, pp. 5–6: ‘Malgré la quantité de belles recherches qui ont paru dans ces derniers tems sur la cause des irregularités de la Lune, il faut convenir que la Theorie de l‘attraction, sur la quelle ces recherches sont toutes fondées, n’a pas encore reçu toute la lumiere qu’elle devoit tirer d’un sujet aussi important. Un des points les plus essentiels qu’il embrasse, la revolution de l’Apogee de la Lune, a cause des discussions très delicates & a donné l’occasion de proposer des supplemens à la Loi generale des Forces. A la verité l’un des Mathematiciens qui avoit eû recours à ces expediens s’est retracté, & a annoncé qu’il avoit trouvé le moien de tirer de sa Theorie le vrai mouvement de l’Apogée sans employer d’autre force que cell qui suit la proportion inverse du quarré des distances.’

^70^
Leonhard Euler to Alexis Clairaut, 29 June 1751, [36], p. 211: ‘Car il est bien certain que ce n’est que depuis cette decouverte, qu’on puisse regarder la loi d’attraction reciproquement proportionelle aux quarrés des distances comme solidement établie, d’où depend cependant toute la Théorie de l’Astronomie.’

^71^
For accounts of Jacquier’s and Le Seur’s scientific activity in Rome see [39] and [40].

**^72See^ :**[3], I, pp. 86–102. See [4], p. 356; [33], p. 157; [41], p. 162.

**^73See^ :**[7], p. 130; [18], p. 90.

**^74See^ :**[4], pp. 341, 355–359; [33], pp. 154–156. The use of asterisks to indicate Calandrini’s authorship was introduced in volume 2, but it was dropped in the second part of volume 3 after it was seen to imply a higher level of scholarly expertise on his part compared with the annotations of Le Seur and Jacquier.

**^75See^ :**[3], II, pp. 364–386.

^76^
See for example, Alexis Clairaut to Gabriel Cramer, 13 April 1744, Bibliothèque de Genève, Ms Suppl. 359, f. 5–6; [16], p. 217, and [33], p. 160.

^77^
*Neue Zeitungen von Gelehrten Sachen*, 17 July 1741, p. 507: ‘Herr Gabriel Cramer hat gewiesen, daß Newtons Beweis nicht schließe, indem er einen von dem newtonischen ganz unterschiedenen Satz auf eben die Art, wie Newton den seinigen beweiset. Die beyden Patres rücken Cramers Ausführung von Wort zu Wort ein. Sie selbst glauben, Newtons Ausspruch sey wahr, aber von ihm nicht genug bewiesen, und bemühen sich daher, einen neuen Beweis davon zu geben.’

^78^
Daniel Bernoulli to Gabriel Cramer, 27 November 1743, Bibliothèque de Genève, Ms Suppl. 384, f. 9v: ‘si cela est vrai j’aurois souhaité qu’on m’en eut averti, car j’aurois pu faire plusieurs additions a ma piece pour faire voir le merveilleux accord entre mes formules et les experiences ou observations, dont je n’etois pas informé auparavant.’

^79^
The three tracts are found in [3], III, 1, pp. 132–374.

^80^
Daniel Bernoulli to Leonhard Euler, 13 June 1744 [42], p. 610/613: ‘In Genff hat man *Principia Newtoni* getruckt in 3 *tomis*, mit überaus viel guten *notis* , worin Ew. HEdgb. sehr offt citiert werden: Man hat auch Dero piece *de aestu maris*, samt des H. Mac Laurins und meiner, *de verbo ad verbum* gantz inseriert.’

^81^
Johann II Bernoulli to Gabriel Cramer, 10 April 1743, Bibliothèque de Genève, Ms Suppl. 384, f. 35r: ‘[…] Ce sont aussi les sentimens de mon Père qui m’a chargé de vous en assurer de même que de toute son amitié. Un ami m’a prié de vous demander, Monsieur, ce que c’étoit que les Princip. Phil. nat. Newt. cum Comment P. P. Le Seur et Jaquier qui ont été imprimés à Genev et si ce livre valoit qu’on l’achetât.’

^82^
See [5], p. 419.

^83^
Rather bitterly, von Thun described the sojourn as ‘Erziehungsexil’. See his letter to Louise Dorothea of 25 September 1744, Thüringisches Statsarchiv Gotha, Geheimes Archiv, E XIII a 25, f. 10v; [25], pp. 46–47. As we learn from a letter to Euler, Cramer first met Frederick-Louis during an outing in the countryside in the summer of 1745. See Gabriel Cramer to Leonhard Euler, 30 August 1746; [11], p, 223.

^84^
See von Thun to Louise Dorothea, 10 December 1747, Thüringisches Statsarchiv Gotha, Geheimes Archiv, E XIII a 26, f. 176r-179r; [43], p. 117–118.

^85^
See von Thun to Louise Dorothea, 8 October 1747, Thüringisches Statsarchiv Gotha, Geheimes Archiv, E XIII a 26, f. 132r–135r; [43], pp. 93–95.

**^86See^ :**[44]. See [23], pp. 138–139, and [6], p. 37.

**^87See^ :**[45], p. 19. On Jacquier’s relation to Mme. Du Châtelet see also [4], pp. 338–339.

^88^
Gabriel Cramer to Johann II Bernoulli, 11 June 1745, Universitätsbibliothek Basel UAH L 1a, p. 783–784: ‘Monsieur le Baron de Thun, ami particulier de M. de Maupertuis, cherche partout la dernière Edition de Londres des Principes de Newton. C’est pour Madame la Marquise du Chatelet, qui en a affaire. Nous ne le trouvons point ici. J’en avois, mais je l’ai fournie a Barillot, pour l’impression qu’il a faite de cet ouvrage avec le Commentaire des P.P. Leseur & Jaquier. J’ai pensé mon cher Monsieur, qu’il ne seroit pas impossible d’en trouver un Exemplaire chés vous. Et si je ne me trompe fort Monsieur votre Père en doit avoir un. Nous serions très oblige s’il pouvoit le resoudre a s’en faire. Je puis aisement lui en fain venir un autre de Londres; a moins qu’il n’aime mieux qu’on lui en paie la valeur, ou qu’il ne voulut reçevoir en échange un Exemplaire de l’Edition de Genève avec le commentaire si Monsieur votre Père, ou quelque autre Possesseur de cette Edition, vouloit bien avoir cette complaisance; vous auriez celle de prendre soin de le faire transmettre a Paris a Madame la M. du Chatelet, par la voie le plus convenable & le plus courte.’

^89^
Johann II Bernoulli to Cramer, 16 June 1745, Bibliothèque de Genève, Ms Suppl. 362, f. 3–4.

^90^
Du Châtelet to Johann II Bernoulli, 6 September 1746, Universitätsbibliothek Basel UBH L 1a 684, pp. 511–514, 517–520: ‘vous avés deja eü la bonté de me procurer un neuton de l’edition de 1726 que ie n’aurais pu traduire sans vous faute d’avoir la bonne edition. J’ay encore recours à vous pour vous prier de me faire avoir un jaquier, c’est à M. bousquet à Lausane qu’il faut s’adresser […] ie vous aurai Monsieur la plus grande obligation car cela me manque absolument et i’en ai un besoin extreme.’ In the haste of writing this letter, Du Châtelet mistakenly refers to ‘two volumes’ of volume three instead of two parts.

^91^
Ulrich von Thun to Duchess Louise Dorothea, 17 April 1747, Thüringisches Staatsarchiv Gotha, Geheimes Archiv, E XIII a 26, f. 45r; [43], p. 38.

^92^
The tract was [46]. See Leonhard Euler to Gabriel Cramer, 21 May 1743; [11].

^93^
See Gabriel Cramer to Leonhard Euler, 18 June 1743; [11], p. 185.

**^94See^ :**[47].

^95^
See Gabriel Cramer to Leonhard Euler, 30 September 1744; [11]. He describes the second reason as being a little more delicate than the first: ʻLa seconde un peu plus délicate, cʼest que jʼai aussi composé moi même il y a 4 ou 5 ans, un petit Essai qui roule sur la même matière que la seconde partie de votre *Traité*.ʼ

^96^
Gabriel Cramer to Nicolaus I Bernoulli, 4 August 1750, Universitätsbibliothek Basel 1a 22:1, No. 71: ‘Il y a soit longtems qu’il devoir paroitre, sans divers contretems forces tantôt de ma part, tantôt de celle de divers libraires, qui en ont retardé la publication jus qu’à aujourd hui.’

^97^
[48], p. xi: ‘J’aurois tiré une grande utilité de *l’Introduction à l’Analyse des infiniment petits* de Mr. Euler, si ce Livre m’avoit été plûtôt connu. Son objet étant presque le même que le mien, il n’est pas surprenant que nous nous soions souvent rencontré dans les Conclusions.’ See also [10], pp. 8–9.

^98^
See [49] and [10], especially pp. 19–29.

^99^
See [18], pp. 95–97.

**^100See^ :**[50], pp. 139–162 (with six tables). See [51], pp. 109–117.

**^101See^ :**[48], pp. viii–ix: ‘Il est facheux que Mr. Newton se soit contenté dʼétaler ses découvertes sans y joindre les Démonstrations, & quʼil ait préféré le plaisir de se faire admirer à celui dʼinstruire.ʼ

**^102See^ :**[51], pp. 111–112.

^103^
See [48], p. 658; [8], pp. 80–85.

^104^
See [52], pp. 56, 97, 103, 105–107, 112. [53], had earlier drawn attention to Leibniz’s anticipation of Cramer’s discovery.

**^105See^ :**[8], p. 16.

^106^
Gabriel Cramer to Alexis Clairaut, 14 September 1750 (minute), Bibliothèque de Genève, Ms Fr. 657b, f. 76–77; [16], p. 231: ‘Mr Calandrin me charge de vous dire qu’il est très content de la façon dont vous avez parlé de lui dans le Memoire de 1745 (nous ne les avons vus que depuis peu). C’est un homme perdu pour es Mathématiques. Ses devois de Conseiller l’occupent entièrement, et il ne manquera point de s’y distinguer.’

^107^
Daniel Bernoulli to Jean Jallabert, undated; [55], p. 118: ‘J’ai perdu un intime Ami. Votre ville et notre Suisse ont perdu un de leurs plus beaux ornemens, et toute l’Europe un savant de premier Ordre, né pour augmenter et pour perfectionner les Sciences […] C’était non seulement un illustre, mais encore un aimable Savant.’
